# Etiology and clinical characteristics of neonatal sepsis in different medical setting models: A retrospective multi-center study

**DOI:** 10.3389/fped.2022.1004750

**Published:** 2022-10-05

**Authors:** Yuanqiang Yu, Qingyi Dong, Suping Li, Huaxue Qi, Xin Tan, Hong Ouyang, Jintao Hu, Wen Li, Tao Wang, Yonghui Yang, Xiaoyun Gong, Xiaori He, Pingyang Chen

**Affiliations:** ^1^ Department of Pediatrics, The Second Xiangya Hospital, Central South University, Changsha, China; ^2^ Laboratory of Neonatal Disease, Institute of Pediatrics, Central South University, Changsha, China; ^3^ Department of Neonatology, Children's Medical Center, The Second Xiangya Hospital, Central South University, Changsha, China; ^4^ Department of Neonatology, Hunan Provincial Maternal and Child Health Care Hospital, Changsha, China; ^5^ Department of Neonatology, Changsha Hospital for Maternal / Child Health Care, Changsha, China; ^6^ Department of Pediatrics, The First Hospital of Changsha, Changsha, China; ^7^ Department of Neonatology, Xiangtan Central Hospital, Xiangtan, China

**Keywords:** pathogens, antimicrobial resistance, clinical characteristics, neonatal sepsis, medical setting

## Abstract

**Objective:**

General hospitals admit lower gestational age neonates than maternal and child health care centers, therefore associated with a higher morbidity and mortality. This study aimed to assess the etiology and clinical characteristics of neonatal sepsis in different medical setting models.

**Methods:**

Neonates admitted to 5 tertiary medical centers, including one national general hospital, two maternal and child health care hospitals and two regional general hospitals, in central-south China with culture-proven sepsis between January 2010 and December 2019 were included in the study. We compared maternal and neonatal characteristics, pathogen distribution, treatment and neonatal outcomes among 3 different medical setting models in this retrospective cohort.

**Results:**

We identified 757 episodes of culture-proven sepsis in 757 neonates. The predominant pathogens were coagulase-negative staphylococci, *Klebsiella pneumoniae*, *Escherichia coli* and Group B streptococci. A total of 683 neonates with detailed information were involved in further comparison; 54.6% were from the national general hospital, 35.9% were from the maternal and child health care hospital, and 9.5% were from the regional general hospital. Neonates in national and regional general hospitals had significantly lower gestational age and birthweight (*P* < 0.001). Patterns of pathogen distribution were different among these medical setting models. Early-onset sepsis was more common in maternal and child health care hospitals (61.4% vs. 42.1% vs. 46.7%, *P* < 0.001), while hospital-acquired late-onset sepsis was more common in national and regional general hospitals (32.7% vs. 33.3% vs. 11.4%, *P* < 0.001). The proportion of complications or comorbidities of neonates in maternal and child health care hospitals were significantly lower than neonates in national and regional general hospitals (*P* < 0.001). The case fatality rate was significantly higher in regional general hospitals (10.8% vs. 3.2% vs. 0.8%, *P* = 0.001).

**Conclusion:**

We report distinct patterns of clinical characteristics, pathogens and outcomes in patient subgroups with neonatal sepsis from national general hospital, maternal and child health care hospital and regional general hospital. It might have some implications for improvement of prevention, management and empirical antibiotic use in neonatal sepsis in different setting models, especially in resource-limited settings from middle and low-income countries.

## Introduction

Sepsis remains a common and frequently fatal condition among neonates globally (1). In Asia, the incidence was higher than in resource-rich countries (2). The pattern of pathogen distribution in Asian settings was similar to that in industrialized countries, yet the important difference was the burden of non-fermenting Gram-negative bacteria, and high level of antibiotic resistance (3). The increasing number of multidrug-resistant Gram-negative micro-organisms is calling for thorough and efﬁcient surveillance strategies and appropriate treatment regimens (4). Neonates in middle and low-income countries is being focused on with novel evidence-based treatment options that are available worldwide (5).

Maternal infection or colonization, total parenteral nutrition, and peripherally inserted central catheter placement were most relevant risk factors of neonatal sepsis (6, 7). Several guidelines had provided a framework for the development of evidence-based approaches to sepsis management (8–10). The application of such strategies like early-onset sepsis (EOS) risk calculator and quality improvement program in resource-limited settings has shown effectiveness in reducing neonatal infection and unnecessary antibiotic exposure (11, 12). However, surveillance of infections with population-level measures in the health facilities and community is still insufficient in middle and low-income countries (13). In China, most settings are still short of high-quality medical staffs, life-sustaining medical devices and sepsis management measures such as intrapartum antibiotic prophylaxis (IAP), application of EOS risk calculator or care bundles and multidisciplinary antimicrobial stewardship in neonatal units.

General hospitals are responsible for treatment of diseases and admit patients with severe conditions, while maternal and child health care hospitals are charged with basic labor services in China. We hypothesized that neonatal sepsis in resource-limited medical setting models including national general hospital, maternal and child health care hospital and regional general hospital represented distinct entities characterized by different pathogen distribution and clinical characteristics. Pathogen surveillance strategies and empirical antibiotic choices, as well as infection, prevention and control measures, may be different in these setting models. This study may provide novel perspective for better improvement in neonatal sepsis management in different medical setting models from middle and low-income countries.

## Methods

### Population

This multi-center retrospective study included 5 tertiary medical centers with neonatal units from central-south China. Among these hospitals, one was the national general hospital (Second Xiangya Hospital, Central South University, 50 beds), two were maternal and child health care hospitals (Hunan Provincial Maternal and Child Health Care Hospital, 150 beds; Changsha Hospital for Maternal/Child Health Care, 70 beds) and two were regional general hospitals (The First Hospital of Changsha, 20 beds; Xiangtan Central Hospital, 50 beds). Four hospitals were from Changsha and one was from Xiangtan. All of them were perinatal centers with neonatal intensive care, providing labor service and neonatal health care for most population in central-south China.

Between January 1, 2010 and December 31, 2019, neonates admitted to these 5 neonatal units and diagnosed culture-proven sepsis were involved in this study. They were followed until death or cured from sepsis and discharge. Neonates were eligible if they developed culture-proven sepsis before 28 days of life (when gestational age ≥37 weeks) or 44 weeks corrected age (when gestational age <37 weeks). An episode of sepsis was defined as one or more positive culture gained from blood, cerebrospinal fluid (CSF) or other sterile sites when clinically indicated. A blood culture was often collected if patients had clinical signs and sepsis was suspected. The date of sepsis onset was defined by the date of first blood culture collection. The samples were taken onsite at each hospital, and a microbiologist was consulted to assist with interpretation of the culture results. Clinical Laboratory Standards Institute (CLSI) guidelines were followed in antimicrobial susceptibility testing (14). Repeatedly positive samples were considered to represent the same episode of infection unless they occurred more than 21 days after the last positive culture result.

Cases were excluded based on the following criteria: (1) cultures considered as contaminants with clinical indication [e.g., coagulase-negative staphylococci (CoNS) in the absence of clinical signs], and antimicrobial therapy was discontinued in less than 5 days; (2) neonates discharged before cured or transferred to other hospitals.

The study was approved by the Ethics Committee of the Second Xiangya Hospital of Central South University and recognized by all participating centers. Data on demographics, maternal and neonatal characteristics, pathogen distribution, antimicrobial resistance (AMR) of predominant isolates, treatment and neonatal outcomes were recorded retrospectively from medical information systems. Data were collected using a standardized questionnaire completed by clinicians for each episode of sepsis.

### Definitions

We defined early-onset sepsis (EOS) by onset in the ﬁrst 72 h of life and late-onset sepsis (LOS) by onset more than 3 days. According to the time of sepsis onset, LOS was divided into hospital-acquired (onset during hospitalization and >48 h after admission) and community-acquired (onset ≤48 h after admission from home).

Premature rupture of membranes was deﬁned as rupture of membranes >24 h prior to delivery. Neonatal bacterial meningitis was defined as positive CSF culture, Gram staining, or neutrophilic leukocytosis, with or without low sugar (less than 50% of plasma glucose level) and high protein content in CSF samples. Neonatal necrotizing enterocolitis (NEC) was defined as ≥ stage 2 according to Bell criteria (15). Intraventricular hemorrhage (IVH) was defined as ≥ grade 3 according to Papile's criteria (16). Bronchopulmonary dysplasia (BPD) was defined as mechanical ventilation or oxygen dependency at 36 weeks of postnatal age or discharge (17). Retinopathy of prematurity (ROP) was defined as ≥ stage 3 according to the International Classification of ROP (18).

### Statistical analysis

We calculated the frequency for categorical variables, and differences in categorical variables were compared using Pearson *χ*^2^ test or Fisher's exact test, where appropriate. For continuous variables with normally distributed data, we calculated the mean ± standard deviation. Continuous variables were compared using one-way ANOVA test. The homogeneity test of variance was performed. We used Welch's ANOVA test for continuous variables with unequal variance. For continuous variables with data not normally distributed, we calculated the median and interquartile range. Accordingly, continuous variables were compared using the Kruskal-Wallis test. A 2-sided of *P* value of <0.05 was considered statistically significant. Statistical analysis was performed using SPSS 26.0 (SPSS Inc, Chicago, IL, USA).

## Results

### Pathogen distribution and antimicrobial resistance

During the 10-year study period, 757 neonates with 757 episodes of culture-proven sepsis met eligibility criteria and were involved in the study. A total of 788 isolates were identified in 5 settings. Therein, 469 isolates (59.5%) were identified in the national general hospital, 250 (31.7%) in maternal and child health care hospitals, and 69 (8.8%) in regional general hospitals. Nearly all isolates were identified from blood culture samples. 16 isolates were identified from CSF samples (including 3 isolates also identified from the blood culture samples) and 5 from other sterile site samples in the national general hospital; 1 isolate was identified both from the blood culture and CSF samples in maternal and child health care hospitals; and 4 isolates were identified from CSF samples (including 1 isolate also identified from the blood culture sample) in regional general hospitals. Multiple organisms were isolated from the same episode in 3.8% (*n* = 29) of neonates. The distribution of pathogens was listed in Table 1. Sepsis were mainly caused by Gram-positive bacteria (*n* = 457, 58.0%). CoNS (*n* = 318, 40.4%), *K. pneumoniae* (*n* = 124, 15.7%), *E. coli* (*n* = 114, 14.5%) and Group B streptococci (GBS) (*n* = 57, 7.2%) were the leading pathogens in all episodes. The most common pathogen isolated was CoNS in EOS episodes (*n* = 115, 35.8%) and in LOS episodes (*n* = 203, 43.5%). *E. coli* (*n* = 57, 17.8%) and GBS (*n* = 38, 11.8%) were also common in EOS. *K. pneumoniae* and *E. coli* were responsible for 20.8% (*n* = 97) and 12.2% (*n* = 57) of episodes in LOS.

**Table 1:** Pathogen distribution of neonatal sepsis in 5 settings from central-south China, 2010–2019.

Pathogens	All isolates (*n*/%)	EOS (*n*/%)	LOS (*n*/%)
**Gram-positive bacteria**	**457/58.0**	**201/62.6**	**256/54.8**
Coagulase negative staphylococci	318/40.4	115/35.8	203/43.5
*Staphylococcus epidermidis*	135/17.1	45/14.0	90/19.3
*Staphylococcus haemolyticus*	42/5.3	24/7.5	18/3.9
*Staphylococcus hominis*	25/3.2	7/2.2	18/3.9
*Staphylococcus saprophyticus*	16/2.0	5/1.6	11/2.4
*Staphylococcus capitis*	4/0.5	0/0.0	4/0.9
*Staphylococcus warneri*	4/0.5	4/1.2	0/0.0
*Staphylococcus chromogenes*	3/0.4	1/0.3	2/0.4
*Staphylococcus cohnii*	2/0.3	2/0.6	0/0.0
*Staphylococcus simulans*	2/0.3	1/0.3	1/0.2
Group B streptococci	57/7.2	38/11.8	19/4.1
*Staphylococcus aureus*	25/3.2	12/3.7	13/2.8
*Enterococcus* species	31/3.9	18/5.6	13/2.8
*Enterococcus faecium*	11/1.4	5/1.6	6/1.3
*Enterococcus faecalis*	17/2.2	11/3.4	6/1.3
*Streptococcus* species	16/2.0	11/3.4	5/1.1
*Streptococcus hemolyticus*	8/1.0	5/1.6	3/0.6
Other Gram-positive bacteria	10/1.3	7/2.2	3/0.6
**Gram-negative bacteria**	**283/35.9**	**108/33.6**	**175/37.5**
*Klebsiella pneumoniae*	124/15.7	27/8.4	97/20.8
*Escherichia coli*	114/14.5	57/17.8	57/12.2
*Serratia* species	12/1.5	4/1.2	8/1.7
*Serratia marcescens*	10/1.3	2/0.6	8/1.7
*Pseudomonas* species	9/1.1	6/1.9	3/0.6
*Pseudomonas aeruginosa*	6/0.8	4/1.2	2/0.4
*Acinetobacter* species	7/0.9	3/0.9	4/0.9
*Acinetobacter baumannii*	3/0.4	2/0.6	1/0.2
*Citrobacter freundii*	3/0.4	1/0.3	2/0.4
*Alcaligenes faecalis*	3/0.4	3/0.9	0/0.0
*Stenotrophomonas maltophilia*	2/0.3	1/0.3	1/0.2
*Enterobacter cloacae*	2/0.3	1/0.3	1/0.2
Other Gram-negative bacteria	7/0.9	5/1.6	2/0.4
**Fungus**	**48/6.1**	**12/3.7**	**36/7.7**
*Candida* species	36/4.6	8/2.5	28/6.0
*Candida glabrata*	8/1.0	2/0.6	6/1.3
*Candida albicans*	10/1.3	0/0.0	10/2.1
*Candida parapsilosis*	6/0.8	0/0.0	6/1.3
*Candida guilliermondi*	3/0.4	1/0.3	2/0.4
Other fungus	12/1.5	4/1.2	8/1.7
**Total**	**788/100.0**	**321/100.0**	**467/100.0**

Tables 2, 3 showed the AMR of predominant pathogens in neonates. Most CoNS isolates were tested resistant to penicillins with or without β-lactamase inhibitors. Nearly all GBS isolates tested were sensitive to penicillin, ampicillin, and vancomycin. *K. pneumoniae* exhibited high resistance to penicillins and cephalosporins. The resistance of *K. pneumoniae* to meropenem was 23.4% and to imipenem was 28.4%. The resistance of *E. coli* to piperacillin/tazobactam was 2.9%, to ceftazidime 14.6% and to meropenem 1.2%. High resistance to ceftriaxone was found in *K. pneumoniae* (78.9%) and *E. coli* (51.4%).

**Table 2:** Antimicrobial resistance in predominant isolates of Gram-positive bacterial neonatal sepsis.

	CoNS, *n*/*N* (%)	GBS, *n*/*N* (%)
Penicillin	225/239 (94.1)	1/57 (1.8)
Oxacillin	208/267 (77.9)	-
Ampicillin	172/178 (96.6)	1/44 (2.3)
Ampicillin/sulbactam	26/29 (89.7)	-
Amoxicillin/clavulanate	169/240 (70.4)	-
Piperacillin/tazobactam	19/24 (79.2)	-
Cefazolin	50/81 (61.7)	-
Cefoxitin	121/134 (90.3)	-
Cefuroxime	33/63 (52.4)	-
Cefotaxime	21/24 (87.5)	0/11 (0.0)
Ceftriaxone	42/48 (87.5)	0/39 (0.0)
Cefoperazone/sulbactam	19/24 (79.2)	-
Cefepime	21/24 (87.5)	0/21 (0.0)
Chloromycetin	5/38 (13.2)	0/8 (0.0)
Erythromycin	208/290 (71.7)	44/53 (83.0)
Azithromycin	75/102 (73.5)	11/14 (78.6)
Clindamycin	87/224 (38.8)	41/55 (74.5)
Trimethoprim/sulfamethoxazole	103/287 (35.9)	1/1 (100.0)
Ciprofloxacin	105/282 (37.2)	2/3 (66.7)
Moxifloxacin	6/79 (7.6)	1/2 (50.0)
Levofloxacin	20/71 (28.2)	15/53 (28.3)
Norfloxacin	6/13 (46.2)	-
Gentamicin	112/288 (38.9)	-
Tobramycin	40/148 (27.0)	-
Amikacin	5/190 (2.6)	-
Tetracycline	47/242 (19.4)	37/38 (97.4)
Tigecycline	0/12 (0.0)	0/2 (0.0)
Minocycline	0/14 (0.0)	-
Linezolid	1/264 (0.4)	0/50 (0.0)
Vancomycin	0/290 (0.0)	0/56 (0.0)
Teicoplanin	0/233 (0.0)	-

CoNS, coagulase negative staphylococci; GBS, group B streptococci. *n*, number of resistant isolates; *N*, number of tested.

**Table 3:** Antimicrobial resistance in predominant isolates of Gram-negative bacterial neonatal sepsis.

	*K. pneumoniae*, *n*/*N* (%)	*E. coli*, *n*/*N* (%)
Ampicillin	103/104 (99.0)	64/92 (69.6)
Ampicillin/sulbactam	93/102 (91.2)	14/94 (14.9)
Amoxicillin/clavulanate	74/107 (69.2)	4/84 (4.8)
Piperacillin	92/102 (90.2)	53/85 (62.4)
Piperacillin/tazobactam	69/116 (59.5)	3/104 (2.9)
Cefazolin	103/110 (93.6)	41/63 (65.1)
Cefoxitin	7/24 (29.2)	0/16 (0.0)
Cefuroxime	30/35 (85.7)	14/22 (63.6)
Cefotaxime	95/99 (96.0)	28/77 (36.4)
Ceftazidime	94/114 (82.5)	15/103 (14.6)
Ceftriaxone	30/38 (78.9)	18/35 (51.4)
Cefoperazone/sulbactam	10/23 (43.5)	2/12 (16.7)
Cefepime	98/115 (85.2)	29/102 (28.4)
Chloromycetin	21/83 (25.3)	5/71 (7.0)
Trimethoprim/sulfamethoxazole	53/111 (47.7)	47/91 (51.6)
Ciprofloxacin	8/109 (7.3)	25/97 (25.8)
Moxifloxacin	2/43 (4.7)	12/40 (30.0)
Levofloxacin	2/112 (1.8)	24/100 (24.0)
Norfloxacin	2/11 (18.2)	2/5 (40.0)
Gentamicin	35/110 (31.8)	34/101 (33.7)
Tobramycin	7/34 (20.6)	6/23 (26.1)
Amikacin	2/113 (1.8)	1/97 (1.0)
Meropenem	22/94 (23.4)	1/82 (1.2)
Imipenem	31/109 (28.4)	1/97 (1.0)
Ertapenem	2/18 (11.1)	0/20 (0.0)
Tetracycline	55/105 (52.4)	53/79 (67.1)
Tigecycline	0/22 (0.0)	0/12 (0.0)
Minocycline	1/10 (10.0)	2/9 (22.2)
Aztreonam	94/111 (84.7)	28/96 (29.2)
Polymyxin B	0/22 (0.0)	0/7 (0.0)

*n*, number of resistant isolates; *N*, number of tested.

### Demographic and clinical characteristics of neonates

In the 5 perinatal centers, a total of 683 neonates with detailed information of clinical characteristics were involved in further comparison. 384 (56.2%) of the neonates were boys. The mean gestational age was 36.5 ± 4.0 weeks, and the mean birthweight was 2704 ± 901 g. The median postnatal age at sepsis onset was 5 (0–16) days. The average hospital stay was 16 (13–27) days. Twenty-one (3.1%) patients died from sepsis before discharged, wherein 9 died from *E. coli* infection, 5 from CoNS infection and 5 from *K. pneumoniae* infection.

Of the 683 neonates with culture-proven sepsis, 373 (54.6%) were from the national general hospital, 245 (35.9%) were from the maternal and child health care hospital, and 65 (9.5%) were from the regional general hospital. Neonates in national and regional general hospitals had significantly lower gestational age and birthweight (*P* < 0.001). Patients transferred from other hospitals accounted for 18.2% and 12.3% in national and regional general hospitals, respectively. 41.2% of the patients in maternal and child health care hospitals were from community. Among the episodes of sepsis, early-onset sepsis was more common in maternal and child health care hospitals (61.4% vs. 42.1% vs. 46.7%, *P* < 0.001), while hospital-acquired late-onset sepsis was more common in national and regional general hospitals (32.7% vs. 33.3% vs. 11.4%, *P* < 0.001). Patterns of pathogen distribution were different in these medical setting models (Table 4). The distribution of GBS was significantly higher in maternal and child health care hospitals, while the distribution of *K. pneumoniae* and *Candida* spp were significantly higher in the national general hospital (*P* < 0.001). The case fatality rate was significantly higher in regional general hospitals (10.8% vs. 3.2% vs. 0.8%, *P* = 0.001).

**Table 4:** Baseline demographic and clinical characteristics of neonates with culture-proven sepsis.

	Total (*n* = 683)	National general hospital (*n* = 373)	Maternal and child health care hospital (*n* = 245)	Regional general hospital (*n* = 65)	*P* value*
Male sex	384 (56.2)	212 (56.8)	139 (56.7)	33 (50.8)	0.648
Gestational age, weeks	36.5 ± 4.0	35.8 ± 4.1	37.7 ± 3.2	35.8 ± 4.4	<0.001
Gestational age groups					
<28 weeks	15 (2.2)	11 (3.0)	2 (0.8)	2 (3.1)	<0.001
28–31 weeks	104 (15.3)	73 (19.6)	15 (6.1)	16 (25.0)	
32–36 weeks	163 (24.0)	104 (28.0)	47 (19.3)	12 (18.8)	
≥37 weeks	398 (58.5)	184 (49.5)	180 (73.8)	34 (53.1)	
Birthweight, g	2704 ± 901	2559 ± 941	2964 ± 772	2551 ± 908	<0.001
Birthweight groups					
<1,000 g	15 (2.2)	11 (2.9)	2 (0.8)	2 (3.1)	<0.001
1,000–1,499 g	80 (11.7)	55 (14.7)	13 (5.3)	12 (18.8)	
1,500–2,499 g	154 (22.6)	97 (26.0)	42 (17.1)	15 (23.4)	
≥2,500 g	433 (63.5)	210 (56.3)	188 (76.7)	35 (54.7)	
SGA	56 (8.2)	39 (10.5)	12 (4.9)	5 (7.8)	0.048
Median postnatal age at sepsis onset, days	5 (0–16)	7 (2–18)	3 (0–11)	7 (2–14)	<0.001
Patient source					
Obstetrics department of the same hospital	378 (55.3)	200 (53.6)	141 (57.6)	37 (56.9)	<0.001
Transferred from other hospitals	79 (11.6)	68 (18.2)	3 (1.2)	8 (12.3)	
Community	226 (33.1)	105 (28.2)	101 (41.2)	20 (30.8)	
Infection type					
EOS	286 (49.3)	134 (42.1)	124 (61.4)	28 (46.7)	<0.001
Hospital-acquired LOS	147 (25.3)	104 (32.7)	23 (11.4)	20 (33.3)	
Community-acquired LOS	147 (25.3)	80 (25.2)	55 (27.2)	12 (20.0)	
Pathogen**					
Gram-positive bacteria	406 (59.4)	210 (56.3)	165 (67.3)	31 (47.7)	0.003
CoNS	278 (40.7)	179 (48.0)	84 (34.3)	15 (23.1)	<0.001
GBS	58 (8.5)	9 (2.4)	41 (16.7)	8 (12.3)	<0.001
Other Gram-positive bacteria	75 (11.0)	24 (6.4)	43 (17.6)	8 (12.3)	<0.001
Gram-negative bacteria	251 (36.7)	145 (38.9)	78 (31.8)	28 (43.1)	0.111
*K. pneumoniae*	112 (16.4)	97 (26.0)	9 (3.7)	6 (9.2)	<0.001
*E. coli*	103 (15.1)	29 (7.8)	55 (22.4)	19 (29.2)	<0.001
Other Gram-negative bacteria	40 (5.9)	22 (5.9)	15 (6.1)	3 (4.6)	0.971
*Candida* species	29 (4.2)	26 (7.0)	2 (0.8)	1 (1.5)	<0.001
Hospital stay, days	16 (13–27)	20 (14–34)	13 (11–16)	21 (12–34)	<0.001
Case fatality	21 (3.1)	12 (3.2)	2 (0.8)	7 (10.8)	0.001

SGA, small for gestational age; EOS, early-onset sepsis; LOS, late-onset sepsis; CoNS, coagulase negative staphylococci; GBS, group B streptococci. Categorical variables are presented as frequencies (%), continuous variables as mean ± standard deviation or median (interquartile range). Percentages are based upon available data for each variable.

* *P* value from *χ*^2^ test or Fisher exact test for categorical variables and from ANOVA test or Kruskal–Wallis test for numerical variables.

** All neonates experienced one episode of infection. Multiple organisms were isolated from the same episode in 3.8% (*n* = 26) of neonates.

### Maternal characteristics

The maternal characteristics of the neonates with culture-proven sepsis are shown in Table 5. Mothers in maternal and child health care hospitals had significantly higher proportion of chorioamnionitis (*P* = 0.002), higher proportion of premature rupture of membrane (PROM) (*P* = 0.013), and lower proportion of using intrapartum antibiotics (*P* < 0.001) than in national and regional general hospitals.

**Table 5:** Maternal characteristics of neonates with culture-proven sepsis.

	Total (*n* = 683)	National general hospital (*n* = 373)	Maternal and child health care hospital (*n* = 245)	Regional general hospital (*n* = 65)	*P* value*
Mother's age, year	29.7 ± 4.7	29.9 ± 4.7	29.5 ± 4.4	29.0 ± 5.1	0.333
Cesarean section	295 (43.3)	192 (51.5)	84 (34.4)	19 (29.7)	<0.001
Maternal hypertension	60 (8.8)	41 (11.0)	15 (6.1)	4 (6.3)	0.089
Maternal diabetes	124 (18.2)	68 (18.2)	48 (19.7)	8 (12.7)	0.442
Chorioamnionitis**	28 (4.1)	8 (2.1)	19 (7.8)	1 (1.6)	0.002
History of infection during pregnancy	165 (24.8)	110 (29.8)	38 (15.6)	17 (33.3)	<0.001
Maternal intrapartum antibiotics	163 (25.3)	99 (28.8)	39 (16.0)	25 (43.9)	<0.001
Premature rupture of membrane	236 (34.8)	112 (30.2)	102 (41.8)	22 (34.9)	0.013
Fetal distress	79 (11.7)	47 (12.7)	23 (9.4)	9 (14.8)	0.348

Categorical variables are presented as frequencies (%), continuous variables as mean ± standard deviation. Percentages are based upon available data for each variable.

* *P* value from *χ*^2^ test or Fisher exact test for categorical variables and from ANOVA test for numerical variables.

** Including clinical and histologic chorioamnionitis. Clinical chorioamnionitis was defined by the presence of maternal fever >38.0°C during labor with at least 2 of the following criteria: uterine tenderness (without another cause), fetal tachycardia, maternal leukocytosis, maternal heart rate >100 bpm, foul-smelling amniotic fluid.

### Complications or comorbidities

The proportion of complications or comorbidities of neonates in maternal and child health care hospitals were significantly lower than neonates in national and regional general hospitals (*P* < 0.001) (Table 6). Neonates in regional general hospitals showed significantly higher rates of pneumonia (*P* < 0.001), meningitis (*P* < 0.001), disseminated intravascular coagulation (DIC) (*P* = 0.045), and multiple organ failure (MOF) (*P* < 0.001).

**Table 6:** Complications or comorbidities of neonates with culture-proven sepsis.

	Total (*n* = 683)	National general hospital (*n* = 373)	Maternal and child health care hospital (*n* = 245)	Regional general hospital (*n* = 65)	*P* value*
Complications or comorbidities	483 (70.7)	289 (77.5)	138 (56.3)	56 (86.2)	<0.001
Pneumonia	264 (38.7)	136 (36.5)	85 (34.7)	43 (66.2)	<0.001
Meningitis	77 (11.3)	45 (12.1)	14 (5.7)	18 (27.7)	<0.001
NEC	16 (2.3)	12 (3.2)	2 (0.8)	2 (3.1)	0.106
IVH	72 (10.5)	57 (15.3)	8 (3.3)	7 (10.8)	<0.001
PPHN	10 (1.5)	4 (1.1)	3 (1.2)	3 (4.6)	0.106
BPD	29 (4.2)	20 (5.4)	6 (2.4)	3 (4.6)	0.225
ROP	25 (3.7)	22 (5.9)	2 (0.8)	1 (1.5)	0.002
DIC	8 (1.2)	4 (1.1)	1 (0.4)	3 (4.6)	0.045
MOF	10 (1.5)	3 (0.8)	1 (0.4)	6 (9.2)	<0.001

NEC, neonatal necrotizing enterocolitis; IVH, intraventricular hemorrhage; PPHN, persistent pulmonary hypertension of the newborn; BPD, bronchopulmonary dysplasia; ROP, retinopathy of prematurity; DIC, disseminated intravascular coagulation; MOF, multiple organ failure. All neonates were performed neuroimaging and eye examinations during hospitalization. Categorical variables are presented as frequencies (%). Percentages are based upon available data for each variable.

* *P* value from *χ*^2^ test or Fisher exact test for categorical variables.

### Treatment

Penicillins with β-lactamase inhibitors (61.0%), third-generation cephalosporins (33.9%) and carbapenems (31.8%) were widely used for directed therapy of neonates with culture-proven sepsis. Neonates in maternal and child health care hospitals had relatively higher proportion of using penicillins (*P* < 0.001) and lower proportion of using carbapenems (*P* = 0.002) than in national and regional general hospitals. Neonates in maternal and child health care hospitals had shorter duration of antibiotic treatment (*P* < 0.001). Neonates with suspected sepsis had higher proportion of adequate empiric antibiotic treatment in the national general hospital (*P* = 0.001).

Patients in national and regional general hospitals had significantly higher proportion of additional medical support, including blood transfusion, intravenous immune globulin (IVIG) and respiratory support than in maternal and child health care hospitals (*P* < 0.001) (Table 7).

**Table 7:** Antibiotic treatment and additional support for neonates with culture-proven sepsis.

	Total (*n* = 683)	National general hospital (*n* = 373)	Maternal and child health care hospital (*n* = 245)	Regional general hospital (*n* = 65)	*P* value*
Main antibiotic use					
Penicillins	75 (11.2)	10 (2.7)	54 (23.4)	11 (16.9)	<0.001
Penicillins + β-lactamase inhibitors	408 (61.0)	276 (74.0)	106 (45.9)	26 (40.0)	<0.001
Second-generation cephalosporins	34 (5.1)	10 (2.7)	23 (10.0)	1 (1.5)	<0.001
Third-generation cephalosporins	227 (33.9)	117 (31.4)	86 (37.2)	24 (36.9)	0.290
Third-generation cephalosporins + β-lactamase inhibitors	46 (6.9)	12 (3.2)	20 (8.7)	14 (21.5)	<0.001
Fourth-generation cephalosporins	4 (0.6)	0 (0.0)	0 (0.0)	4 (6.2)	<0.001
Moxalactam	41 (6.1)	41 (11.0)	0 (0.0)	0 (0.0)	<0.001
Carbapenems	213 (31.8)	135 (36.2)	53 (22.9)	25 (38.5)	0.002
Vancomycin	32 (4.8)	10 (2.7)	16 (6.9)	6 (9.2)	0.008
Linezolid	9 (1.3)	1 (0.3)	3 (1.3)	5 (7.7)	<0.001
Metronidazole	25 (3.7)	21 (5.6)	4 (1.7)	0 (0.0)	0.012
Other antibiotic	14 (2.1)	5 (1.3)	6 (2.6)	3 (4.6)	0.129
Length of antibiotic treatment, days	15 (12–21)	18 (14–24)	12 (10–14)	17 (11–24)	<0.001
Antibiotic treatment changed after identification of pathogen and antibiotic susceptibilities	100 (15.3)	39 (10.7)	47 (20.5)	14 (23.7)	0.001
Catecholamines	135 (19.8)	78 (20.9)	43 (17.6)	14 (21.5)	0.550
Blood transfusion	161 (23.6)	128 (34.3)	20 (8.2)	13 (20.0)	<0.001
IVIG	242 (35.4)	180 (48.3)	27 (11.0)	35 (53.8)	<0.001
Respiratory support					
None	431 (63.1)	217 (58.2)	185 (75.5)	29 (44.6)	<0.001
Oxygen threapy	137 (20.1)	104 (27.9)	20 (8.2)	13 (20.0)	
CPAP	41 (6.0)	6 (1.6)	29 (11.8)	6 (9.2)	
Non-invasive ventilation (except CPAP and high frequency ventilation)	16 (2.3)	9 (2.4)	3 (1.2)	4 (6.2)	
Invasive ventilation (except CPAP and high frequency ventilation)	44 (6.4)	32 (8.6)	3 (1.2)	9 (13.8)	
High frequency ventilation	14 (2.0)	5 (1.3)	5 (2.0)	4 (6.2)	

IVIG, intravenous immune globulin; CPAP, continuous positive airway pressure. Categorical variables are presented as frequencies (%), continuous variables as median (interquartile range). Percentages are based upon available data for each variable.

* *P* value from *χ*^2^ test or Fisher exact test for categorical variables and from Kruskal–Wallis test for numerical variables.

## Discussion

This multi-center retrospective study described the pathogen distribution and AMR of predominant isolates in neonatal sepsis from central-couth China between January 1, 2010 and December 31, 2019. We also identified key differences among national general hospital, maternal and child health care hospital and regional general hospital in demographic characteristics, pathogens, maternal and neonatal characteristics, complications and comorbidities, and treatment. These findings provided novel perspective in management of neonatal sepsis in middle and low-income countries.

CoNS, GBS, *K. pneumoniae* and *E. coli* remained to be the predominant pathogens in neonatal sepsis in China (3, 7, 19). The pathogen distribution in EOS and LOS had a similar pattern with in developed countries, as well as in developing countries (20–22). High inter-regional heterogeneity was found in prevalence of these leading isolates, especially GBS (7). Our results showed a remarkable prevalence of CoNS both in EOS and LOS, which was different in pathogen distribution in south China (23). Furthermore, the AMR pattern amongst CoNS and *K. pneumoniae* isolates in our study has changed significantly when compared to previous data in Chinese neonates (24). AMR in *K. pneumoniae* and *E. coli* is growing and has become a serious threat to public health in China (25, 26). However, the presence of multidrug resistant micro-organisms was less valued and reported in neonatal units in China, including isolates of extended-spectrum β-lactamase producing or AmpC β-lactamase producing, and carbapenemase resistant Enterobacterales. The multidrug resistant nature of the predominant Gram negative bacteria will be explored in further studies. We also showed a high rate of antibiotic use in penicillins + β-lactamase inhibitors, third-generation cephalosporins and carbapenems as directed therapy. Empirical treatment choices in Chinese hospitals mostly followed the national guidelines, and were fewer reviewed and updated periodically. The pathogen distribution and antibiotic use showed significant difference in 3 medical setting models in our study. We raise the importance of ongoing surveillance of AMR infection (especially multidrug resistant micro-organisms) and individual empirical antibiotic treatment in different setting models. The detection of multidrug resistance forms is also important, and has implication in treatment and infection control that broader spectrum antimicrobial agents will be used.

National general hospitals represent the highest quality of medical services in different fields, including perinatology. Multiple disciplinary teamwork is becoming a prevalent model for clinical decision making in neonatal units in national general hospitals from China. Therefore, neonates with sepsis could be well managed and followed until discharge. In our study, we showed a relative better prognosis of neonatal sepsis in national general hospital than in regional general hospital. However, as a referral center, the gestational age and birthweight were relatively lower and hospital-acquired LOS was more common than in maternal and child health care hospital. The prevalence of *K. pneumoniae* and *Candida* spp were significantly higher in national general hospital, compared to maternal and child health care hospital and regional general hospital. Underlying conditions like catheter insertion and parenteral nutrition were more frequently seen in national general hospitals, thus increasing the incidence of neonatal LOS (27–29). Quality improvement initiatives using care bundles have proven to decrease infection rates in neonatal intensive care units (12, 30, 31). Other initiatives including implementation of the sepsis risk calculator showed good predictive value in EOS and were associated with the reduction in antibiotic use (11, 32, 33). Recently, efforts had been made in reducing unnecessary antibiotic use, and the relationship with neonatal outcomes and AMR was explored in developed countries (34–37). It is necessary to better prevent neonatal infection or sepsis and reduce AMR in national general hospital to improve neonatal outcomes.

In maternal and child health care hospitals, maternal and newborn care are much more paid attention to. Besides, the communication between the doctors and the parents is fluent and often. Any abnormality expressed from the neonates could be found timely and well treated. In our study, it was shown that neonates with sepsis in maternal and child health care hospital had better outcome than neonates from general hospital. However, it was noted that most cases in neonatal sepsis in maternal and child health care hospital were EOS, and GBS accounted 16.7% in pathogen distribution, significantly higher than in national and regional general hospital. GBS emerges to be the leading pathogen of maternal disease and neonatal sepsis in Chinese population since universal maternal GBS screening (38–40). It is known that maternal GBS colonization is the risk factor of neonatal sepsis, therefore, intrapartum antibiotic treatment has been an important strategy for management of neonatal EOS (8, 41). In the US, the application of IAP might lead to the decrease of infant GBS disease, but the burden of GBS and *E. coli* infection still remained (42–45). Maternal GBS screening and IAP strategies had only been applied in recent years in Chinese hospitals. Our study showed an insufficient application of IAP in maternal and child health care hospital, and the proportion of chorioamnionitis and PROM was higher, compared with neonates with sepsis in general hospital. This highlights the importance of administration of maternal GBS detection and IAP, and further management in newborn infants at risk for GBS disease in maternal and child health care hospital.

Regional general hospitals have perinatal centers responsible for treatment of diseases in pregnancy and infants in different cities. These are also referral centers for neonates with complex diseases or serious condition. It is noted that neonates with culture-proven sepsis in regional general hospital had the case fatality of 10.8%, much higher than neonates in national general hospital and maternal and child health care hospital. As reported, low gestational age and birthweight, as well as high requirements of mechanical ventilation might be the potential influence (46). Furthermore, in our study, *E. coli* was the leading pathogen (29.2%) in regional general hospital, and the proportion was significantly higher than in national general hospital and maternal and child health care hospital. Other studies showed that the prevalence of Gram-negative bacteria was associated with high mortality (47–49). The proportion of complications and comorbidities, especially pneumonia and meningitis, were higher in regional general hospital, which might independently lead to sepsis-attributable mortality (50, 51). It was shown that neonatal infection in very preterm infants was associated with an increased risk of poor postnatal weight growth and cerebral palsy at the age of 5 years (52, 53). Notably, compared with culture-proven sepsis, culture-negative LOS was associated with increased risks of adverse outcomes, including neurodevelopmental impairment (54, 55). Therefore, it has indication for more caution in sepsis management and treatment in regional general hospital, and more attention at follow up.

The strength of this study was the generalization of characteristics of neonatal sepsis in different medical setting models. We found that the etiology and clinical characteristics in sepsis had great differences among national general hospital, maternal and child health care hospital and regional general hospital. The variance in patient source and management in neonatal units might result in such differences. Therefore, we raise the focus on the exclusive management of neonatal sepsis in medical settings when interhospital transfers are frequent in resource-limited countries.

There are still some limitations should be considered. First, specialized children's hospitals without labor services were not involved in the study. Patients in these hospitals are transferred from other hospitals or from community. It is a necessity to generalize clinical characteristics of neonatal sepsis in these hospitals in further studies. Second, it should be mentioned that neonates in general hospital were smaller and more prone to morbidity and mortality, which may result in a risk of bias. Finally, we involved cases between January, 2010 and December, 2019 in 5 settings in central-south China. The sample size was still limited. We would further explore the patterns as a multi-center prospective study in different regions.

In conclusion, we described the pathogen distribution and AMR of predominant isolates in neonatal sepsis from central-south China between January, 2010 and December, 2019. The predominant pathogens were CoNS, *K. pneumoniae*, *E. coli* and GBS. We also provided a detailed comparison of neonatal sepsis in 3 different medical setting models. GBS had a higher proportion in maternal and child health care hospital, while the proportion of *K. pneumoniae* and *Candida* spp were higher in national general hospital. The proportion of complications and comorbidities, as well as the mortality rate, in regional general hospital was higher, compared to national general hospital and maternal and child health care hospital. Therefore, we highlight the distinct strategies for improvement of sepsis management in these setting models (quality improvement initiatives for prevention in national general hospital, maternal GBS screening and IAP strategies in maternal and child health care hospital, and staff training in treatment and follow up in regional general hospital). Also, neonatal units in different setting models should have ongoing pathogen surveillance and individual empirical antibiotic treatment choices. These findings may have implications for better management of neonatal sepsis in resource-limited settings from middle and low-income countries.

## Data Availability

The raw data supporting the conclusions of this article will be made available by the authors, without undue reservation.
